# Altered GIP/GLP-1 Secretion Ratio is Associated With Impaired β Cell Function in Humans

**DOI:** 10.1210/clinem/dgaf210

**Published:** 2025-05-12

**Authors:** Gianfranco Di Giuseppe, Giulia Gliozzo, Gea Ciccarelli, Lorenzo Carciero, Michela Brunetti, Laura Soldovieri, Giuseppe Quero, Francesca Cinti, Enrico Celestino Nista, Sara Sofia De Lucia, Antonio Gasbarrini, Sergio Alfieri, Alfredo Pontecorvi, Andrea Mari, Bolette Hartmann, Jens Juul Holst, Andrea Giaccari, Teresa Mezza

**Affiliations:** Endocrinology and Diabetology Unit, University Hospital Agostino Gemelli, 00168 Rome, Italy; Department of Translational Medicine and Surgery, Catholic University of Sacred Heart, 00168 Rome, Italy; Department of Translational Medicine and Surgery, Catholic University of Sacred Heart, 00168 Rome, Italy; Endocrinology and Diabetology Unit, University Hospital Agostino Gemelli, 00168 Rome, Italy; Department of Translational Medicine and Surgery, Catholic University of Sacred Heart, 00168 Rome, Italy; Department of Translational Medicine and Surgery, Catholic University of Sacred Heart, 00168 Rome, Italy; Endocrinology and Diabetology Unit, University Hospital Agostino Gemelli, 00168 Rome, Italy; Department of Translational Medicine and Surgery, Catholic University of Sacred Heart, 00168 Rome, Italy; Endocrinology and Diabetology Unit, University Hospital Agostino Gemelli, 00168 Rome, Italy; Department of Translational Medicine and Surgery, Catholic University of Sacred Heart, 00168 Rome, Italy; Department of Translational Medicine and Surgery, Catholic University of Sacred Heart, 00168 Rome, Italy; Digestive Surgery Unit, University Hospital Agostino Gemelli, 00168 Rome, Italy; Endocrinology and Diabetology Unit, University Hospital Agostino Gemelli, 00168 Rome, Italy; Department of Translational Medicine and Surgery, Catholic University of Sacred Heart, 00168 Rome, Italy; Department of Translational Medicine and Surgery, Catholic University of Sacred Heart, 00168 Rome, Italy; Pancreas Unit, Digestive Diseases Center, University Hospital Agostino Gemelli, 00168 Rome, Italy; Department of Translational Medicine and Surgery, Catholic University of Sacred Heart, 00168 Rome, Italy; Pancreas Unit, Digestive Diseases Center, University Hospital Agostino Gemelli, 00168 Rome, Italy; Department of Translational Medicine and Surgery, Catholic University of Sacred Heart, 00168 Rome, Italy; Pancreas Unit, Digestive Diseases Center, University Hospital Agostino Gemelli, 00168 Rome, Italy; Department of Translational Medicine and Surgery, Catholic University of Sacred Heart, 00168 Rome, Italy; Digestive Surgery Unit, University Hospital Agostino Gemelli, 00168 Rome, Italy; Endocrinology and Diabetology Unit, University Hospital Agostino Gemelli, 00168 Rome, Italy; Department of Translational Medicine and Surgery, Catholic University of Sacred Heart, 00168 Rome, Italy; Institute of Neuroscience, National Research Council, 2-35122 Padua, Italy; Department of Biomedical Sciences, University of Copenhagen, 2200 Copenhagen, Denmark; Novo Nordisk Foundation Center for Basic Metabolic Research, University of Copenhagen, 2200 Copenhagen, Denmark; Endocrinology and Diabetology Unit, University Hospital Agostino Gemelli, 00168 Rome, Italy; Department of Translational Medicine and Surgery, Catholic University of Sacred Heart, 00168 Rome, Italy; Endocrinology and Diabetology Unit, University Hospital Agostino Gemelli, 00168 Rome, Italy; Department of Translational Medicine and Surgery, Catholic University of Sacred Heart, 00168 Rome, Italy; Pancreas Unit, Digestive Diseases Center, University Hospital Agostino Gemelli, 00168 Rome, Italy

**Keywords:** type 2 diabetes, incretin secretion, glucagon-like peptide 1, glucose-dependent insulinotropic peptide, beta cell function

## Abstract

**Introduction:**

The entero-insular axis, mediated by the incretin hormones glucose-dependent insulinotropic polypeptide (GIP) and glucagon-like peptide-1 (GLP-1), is fundamental to maintaining glucose homeostasis. Dysregulation of these hormones’ biology contributes to the pathogenesis of type 2 diabetes (T2D), but the existence of a dysfunctional secretory pattern of incretins toward deterioration of glucose tolerance is still debated. In this study, we evaluate possible impairments in the overall incretin secretion from normal glucose tolerance to overt diabetes, as well as their association with impaired insulin secretion.

**Methods:**

Sixty subjects with an unknown history of T2D who were not on antidiabetic treatments were divided into 3 groups according to oral glucose tolerance test-derived glucose tolerance: normal glucose tolerance (NGT) (n = 23), impaired glucose tolerance (IGT) (n = 16), and diabetes mellitus (DM) (n = 21). All subjects underwent deep metabolic evaluation with a mixed meal test (MMT) and euglycemic hyperinsulinemic clamp. During the MMT, we calculated the GIP/GLP-1 secretion ratio (SR) and the GIP/GLP-1 SR areas under the curve. Parameters of β cell function were obtained by mathematical modeling.

**Results:**

Linear mixed model analysis revealed similar GIP and GLP-1 responses to MMT among the 3 groups, while GIP/GLP-1 SR was reduced in DM subjects compared to NGT and IGT. Further, multiple regression analysis showed a predictive role of GIP/GLP-1 SR on rate sensitivity and standardized insulin secretion at 5 mmol/L.

**Conclusion:**

Our findings demonstrate that, despite similar GIP and GLP-1 secretion, the GIP/GLP-1 SR declines as glucose tolerance deteriorates, reflecting an imbalance in incretin dynamics rather than absolute hormone secretion. This imbalance may indicate early β cell dysfunction and chronic incretin resistance.

In the past few decades, investigations on the entero-insular axis and incretin pathophysiology have gained increasing attention due to their crucial role in the regulation of insulin secretion and glucose metabolism in humans. In this system, the incretin hormones glucose-dependent insulinotropic peptide (GIP) and glucagon-like peptide 1 (GLP-1) are respectively secreted by enteroendocrine K-cells and L-cells in response to the absorption of glucose, amino acids, and lipids by enterocytes (1). As gut-derived endocrine signals directed at the pancreatic islets, GIP and GLP-1 exert their effects by binding specific receptors on β cells and consequently augmenting insulin secretion in response to hyperglycemia (2). Both hormones are responsible for the incretin effect, expressing the potentiation of insulin secretion observed after glucose ingestion compared to an isoglycemic intravenous glucose stimulation (3). In type 2 diabetes (T2D), the incretin effect is severely reduced due to defective β cell responses to both GIP and GLP-1 (4-6). In this context, many studies have been held to identify possible defects in the incretin secretion among deterioration of glucose tolerance, but no substantial differences in the GIP and GLP-1 secretion in response to an oral glucose tolerance test (OGTT) or mixed meal test (MMT) have been identified in T2D patients compared to healthy people (7, 8). Conversely, many studies demonstrated a reduced GIP and GLP-1 islet sensitivity in T2D natural history, including our recent research demonstrating that defects in the insulinotropic effect of incretins can be observed even in nondiabetic subjects during the early phases of the disease, with no major changes in the overall circulating incretin levels (9).

Starting from these assumptions, in this study we explore GIP and GLP-1 responses after a MMT stimulation in a cohort of individuals with different classes of glucose tolerance by analyzing the secretory pattern over time of the 2 hormones together. To pursue this aim, we calculated the GIP/GLP-1 secretion ratio (SR) and explored its role as a possible marker of unbalanced incretin secretion in T2D as well as its association with impaired β cell function and glucose intolerance in an attempt to enhance the understanding of the pathophysiological changes in incretin dynamics across deterioration of glucose tolerance as well as the impaired incretin response observed in T2D.

## Methods

### Study Population

Sixty patients without a known history of diabetes and not on any antidiabetic treatment were recruited and studied at the Centre for Endocrine and Metabolic Diseases—University Hospital Agostino Gemelli. Only patients with normal cardiopulmonary and kidney function, as determined by medical history, physical examination, electrocardiography, estimated glomerular filtration rate (eGFR), and urinalysis, were included. Altered serum lipase and amylase levels were considered exclusion criteria. Patients with severe obesity [body mass index (BMI) > 40], uncontrolled hypertension, and/or hypercholesterolemia were also excluded. All patients underwent an OGTT to determine glucose tolerance; a MMT for the evaluation of glucose, insulin, C-peptide, GIP, and GLP-1 responses; and an euglycemic hyperinsulinemic clamp to evaluate insulin sensitivity.

The clinical and metabolic characteristics of study participants are shown in Table 1.

**Table 1.** Clinical and metabolic characteristics of study participants

Subject characteristics	NGT (n = 23)	IGT (n = 16)	DM (n = 21)	*P*-value
Age (years)	58.6 ± 3.06	59.7 ± 3.78	70.9 ± 2.63	.01*
Sex (F/M)	14/9	8/8	11/10	—
BMI (kg/m**^2^**)	25.4 ± 1.23	25.1 ± 1.06	26.3 ± 0.99	.65
Insulin sensitivity (mg kg^−1^ minute^−1^)	4.81 ± 0.90^*a*^	3.96 ± 0.58	3.63 ± 0.85^*a*^	.05*^,*a*^
Fasting glucose (mg/dL)	89.9 ± 1.99	97.9 ± 4.38	116 ± 5.83	< .001**
Fasting insulin (µUI/mL)	8.86 ± 1.36	9.59 ± 1.82	10.1 ± 2.11	.93
Fasting C-peptide (ng/mL)	2.02 ± 0.21	2.32 ± 0.41	2.12 ± 0.39	.82
eGFR (mL/min/1.73 m^2^)	92.2 ± 4.80	88.8 ± 5.28	88.80 ± 5.01	.40
GIP AUC (pmol·L)	24427 ± 3026	19093 ± 2081	16900 ± 2019	.09
GLP-1 AUC (pmol·L)	5009 ± 617	4545 ± 762	5511 ± 715	.60
Glucagon AUC	2027 ± 234	2559 ± 430	1850 ± 224	.52
GIP/GLP-1 AUC (pmol L^−1^ minute)	1568 ± 226	1272 ± 166	947 ± 145	.02*

Abbreviations: AUC, area under the curve; BMI, body mass index; DM, diabetes mellitus; eGFR, estimated glomerular filtration rate; GIP, glucose-dependent insulinotropic polypeptide; GLP-1, glucagon-like peptide-1; IGT, impaired glucose tolerance; NGT, normal glucose tolerance.

*P < .01; **P < .05.

^a^Post hoc analysis revealed a significant difference between NGT and DM (*P* = .02).

Based on OGTT-derived glucose tolerance, patients were divided into the following 3 groups: NGT (n = 23), IGT (n = 16), and diabetes mellitus (DM; n = 21). Glucose, insulin, and C-peptide levels during OGTT are shown in Supplementary Fig. S1 (10).

### Metabolic Evaluation

#### OGTT

A standard 75 g OGTT was performed with measurement of glucose, insulin, and C-peptide at 0, 30, 60, 90, and 120 minutes after glucose load.

#### MMT

A MMT was performed as previously described (11). Patients were instructed to consume a liquid meal of 830 kcal (107 kcal from protein, 353 kcal from fat, and 360 kcal from carbohydrates) within 15 minutes. Blood samples were drawn twice in the fasting state and at 30-minute intervals over the following 240 minutes (sample time 0, 30, 60, 90, 120, 150, 180, 210, and 240) for the measurement of plasma glucose, insulin, C-peptide, GIP, and GLP-1.

#### Euglycemic hyperinsulinemic clamp

The euglycemic hyperinsulinemic clamp (EHC) test was performed after a 12-hour overnight fast using 40 mIU min^−1^ m^−2^ insulin of body surface, according to DeFronzo and colleagues (12). A primed-constant infusion of insulin was administered (Actrapid HM, Novo Nordisk). The constant rate for the insulin infusion was reached within 10 minutes to achieve steady-state insulin levels; in the meantime, a variable infusion of 20% glucose was started via a separate infusion pump and the rate was adjusted, based on plasma glucose levels measured every 5 minutes, maintaining plasma glucose concentrations at fasting levels. During the last 20 minutes of the clamp procedure, plasma samples from blood drawn at 5- to 10-minute intervals were used to determine glucose and insulin concentrations.

### Biochemical Measurements

At each sampling point, blood was collected in EDTA tubes for insulin and C-peptide measurement. Blood samples for GLP-1 were collected in tubes containing EDTA and a dipeptidyl peptidase-4 inhibitor (Millipore, St. Charles, MO) to prevent the enzymatic degradation of GLP-1 (7-36) and GLP-1 (7-37) and immediately separated in a refrigerated centrifuge (1000 rpm for 10 minutes at 4 °C). Plasma samples were divided into aliquots and stored at −80 °C until analysis. Plasma glucose concentrations were determined bedside using the glucose oxidase technique on a glucose analyzer (Beckman Instruments, Palo Alto, CA). Insulin levels were determined using a commercial radioimmunoassay kit (Medical System; Immulite DPC, Los Angeles, CA). Plasma C-peptide was measured by Auto-DELFIA Fluoroimmunoassay (Wallac, Turku, Finland). Plasma concentrations of GIP and GLP-1 were measured using total GIP (AB_2895085 NL-ELISA, Mercodia, Uppsala, Sweden) and total GLP-1 (AB_2892202 NL-ELISA, Mercodia, Uppsala, Sweden) ELISA.

### Calculations

After OGTT, glucose tolerance was determined according to the American Diabetes Association criteria (Standard of Care 2024): subjects whose 2-hour load glucose was below 140 mg/dL were defined as NGT, subjects whose 2-hour load glucose was 140 to 199 mg/dL were defined as IGT, and subjects whose 2-hour load glucose was equal to or higher than 200 mg/dL were defined as DM.

Moreover, the Matsuda index was derived from OGTT as previously reported (13).

β cell function and total insulin secretion rate (tISR) were assessed using a mathematical model, by C-peptide deconvolution, as previously described (14): the rate sensitivity (RS; pmol m^−2^ mmol^−1^ L) was estimated from OGTT modeling, representing the dependence of the insulin secretion rate on the rate of change in glucose concentration and is related to first-phase insulin secretion (15); the standardized insulin secretion (ISR1; pmol min^−1^ m^−2^) represents the insulin secretion rate at fixed glucose concentrations (5 mmol/L from the dose-response), thus providing a measure of β cell function under basal (ie, normoglycemic) conditions; the β cell glucose sensitivity (βCGS; pmol min^−1^ m^−2^ mM^−1^), describing the slope of the relationship between insulin secretion and glucose concentration, was assessed during OGTT by modeling as previously described.

During MMT, the GIP/GLP-1 SR was calculated as the ratio of GIP to GLP-1 values for each time point during the MMT. During the EHC, whole-body glucose utilization was calculated during the last 30 minutes of the steady-state insulin infusion and was measured as the mean glucose infusion rate (mg kg^−1^ min^−1^).

### Statistics

Categorical variables were described as percentages, while continuous variables were summarized as mean ± SEM. The normality of continuous variables was assessed using the Shapiro-Wilk test and quantile-quantile plots. For normally distributed variables, Levene's test for equality of variances was performed before conducting 1-way ANOVA. For nonnormally distributed variables, comparisons were conducted using the Kruskal-Wallis test. We performed Spearman's or Pearson's correlation analyses, as appropriate, to assess associations between variables. A 2-tailed *P*-value <.05 was considered statistically significant. Differences in glucose, insulin, C-peptide, GIP, GLP-1, and glucagon levels among patients stratified by glucose tolerance were assessed using linear mixed models. To further investigate the associations among variables, 2 hierarchical multiple regression analyses were conducted. In the first model, the outcome variable was RS, while in the second model, the outcome variable was ISR1.

Independent variables were introduced in the regression models stepwise as follows:

- Step 1: BMI (kg/m^2^)
- Step 2: eGFR (a measure of renal function)
- Step 3: Matsuda index (a measure of insulin sensitivity)
- Step 4: GIP/GLP-1 SR AUC

To ensure the absence of multicollinearity among independent variables, variance inflation factor values were examined. All variance inflation factor values were below 5, indicating no significant collinearity issues in the models. Regression models were evaluated using the following statistical indices: *F*-statistic, which assesses the significance of the variance explained by the independent variables; standardized β coefficients, which indicate the strength of each independent variable's effect on the dependent variable; R^2^, representing the percentage of variance in the dependent variable explained by the model; and R^2^ change, indicating the additional variance explained at each step of the hierarchical regression. A 2-tailed *P*-value <.05 was considered statistically significant. All statistical analyses were performed using IBM SPSS Statistics version 29.1 (IBM Corp., 2023).

## Results

### GIP/GLP-1 SR Is Significantly Reduced in Diabetic Subjects

**Figure 1.**
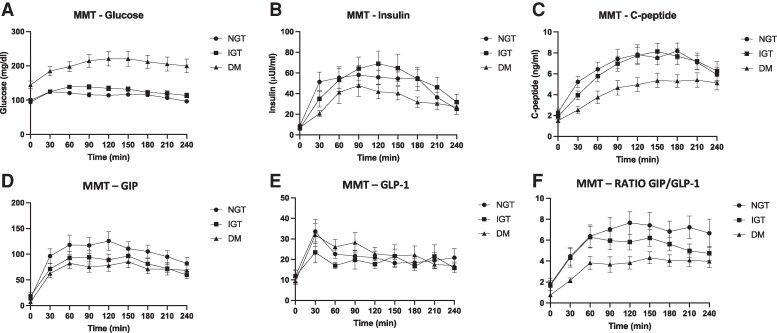
Glucose (A), insulin (B), C-peptide (C), GIP (D), GLP-1 (E), and GIP/GLP-1 SR (F) levels during the mixed meal test in normal glucose tolerance (black circles), impaired glucose tolerance (black squares), and diabetes mellitus (black triangles) groups. Abbreviations: GIP, glucose-dependent insulinotropic polypeptide; GLP-1, glucagon-like peptide-1.

During MMT stimulation, DM subjects displayed increased glucose levels compared to NGT and IGT subjects (Fig. 1A, *P* < .001 for interaction time × glucose tolerance), while C-peptide levels were reduced in DM individuals (Fig. 1C, *P* = .02 for interaction time × glucose tolerance), as expected. No significant differences were observed in insulin responses among the 3 groups (Fig. 1B, *P* = .09 for interaction time × glucose tolerance). A linear mixed model analysis of incretin responses to the MMT showed similar GIP and GLP-1 levels over time among different classes of glucose tolerance (Fig. 1D, *P* = .40 and Fig. 1E, *P* = .2 for interaction time × glucose tolerance, respectively), as confirmed by comparison of GIP and GLP-1 AUCs in the 3 groups (Table 1, *P* = .10 and .60 respectively); similarly, no difference was found in glucagon secretion among the 3 groups [Supplementary Fig. S2 (10), *P* = .34 for interaction time × glucose tolerance].

Furthermore, we calculated the GIP/GLP-1 SR over time, assumed as an index of mutual incretin secretory dynamics, expressing the relationship between secreted levels of GIP and GLP-1 in response to a meal: compared to NGT and IGT people, DM subjects exhibited reduced GIP/GLP-1 SR (Fig. 1F, *P* = .02 for interaction time × glucose tolerance), possibly expressing an unbalanced overall incretin secretion in T2D. Accordingly, the AUC of GIP/GLP-1 SR calculated during the MMT was significantly reduced in DM subjects (Table 1, *P* = .02).

### GIP/GLP-1 SR Predicts Specific β Cell Functional Defects

To investigate the predictive potential of GIP/GLP-1 SR on specific β cell impairments, we conducted hierarchical regression models to assess whether GIP/GLP-1 SR AUC independently predicted β cell function while accounting for potential confounders, including BMI, eGFR, and the Matsuda index, a measure of insulin sensitivity (see *Methods*). Model 1 explained 30.4% of dependent variable variance, where the dependent variable was OGTT-derived RS, an index of the effectiveness of first-phase insulin secretion [Table 2, *F*(4,35) = 5.262 *P* = .002]. Model 2 explained 18.1% of dependent variable variance, where the dependent variable was OGTT-derived standardized insulin secretion at low glucose (ISR1), expressing the ability of β cells to respond to a 5 mmol/L glucose stimulation [Table 3, *F*(4,33) = 3.042 *P* = .03]. By contrast, we observed no correlation between GIP/GLP-1 SR AUC and OGTT-derived tISR (*P* = .98) and β cell glucose sensitivity (βCGS, *P* = .49), as well as with EHC-derived glucose uptake, an index of insulin sensitivity (*P* = .99).

**Table 2.** Multiple regression analysis for predicting rate sensitivity

Variables	Estimate (B)	SE	β	95% CI		*P-*value	R^2^	Adjusted R^2^
				LL	UL			
Step 1								
BMI (kg/m^2^)	19.44	15.82	.20	−12.58	51.47	.23	0.04	0.01
Step 2								
BMI (kg/m^2^)	19.52	16.03	.20	−12.97	52.01	.23		
eGFR	0.58	3.74	0.03	−7.00	8.15	.88	0.04	−0.01
Step 3								
BMI (kg/m^2^)	14.29	16.35	.14	−18.88	47.45	.39		
eGFR	1.49	3.76	.06	−6.14	9.12	.70		
Matsuda index	−17.31	13.04	−.22	−43.76	9.13	.19	0.08	0.01
Step 4								
BMI (kg/m^2^)	6.05	13.84	.06	−22.05	34.15	.67		
eGFR	2.28	3.16	.10	−4.13	8.69	.47		
Matsuda index	−13.76	10.95	−.18	−36.00	8.47	.22		
GIP/GLP-1 SR AUC	0.28	0.07	.55	0.14	0.42	<.001**	0.38	0.30

This table presents unstandardized (B) and standardized (β) regression coefficients, SE, 95% CIs, and significance levels (*P*-values) for each predictor. The dependent variable was oral glucose tolerance test-derived rate sensitivity, an index of first-phase insulin secretion effectiveness. GIP/GLP-1 AUC was the only significant predictor (*P* < .001), while BMI, eGFR, and Matsuda index were not significant. Bold values indicate statistically significant regression coefficients (*P < .05; **P < .01).

Abbreviations: AUC, area under the curve; BMI, body mass index; CI, confidence interval; eGFR, estimated glomerular filtration rate; GIP, glucose-dependent insulinotropic polypeptide; GLP-1, glucagon-like peptide-1; LL, lower limit; SR, secretion ratio; UL, upper limit.

**Table 3.** Multiple regression analysis for predicting ISR1

Variables	Estimate (B)	SE	β	95% CI		*P*-value	R^2^	Adjusted R^2^
				LL	UL			
Step 1								
BMI (kg/m^2^)	−1.27	2.51	−.08	−6.36	3.82	.60	0.007	−0.02
Step 2								
BMI (kg/m^2^)	−1.44	2.45	−.10	−6.42	3.53	.56		
eGFR	−0.98	0.58	−.28	−2.15	0.19	.10	0.08	0.03
Step 3								
BMI (kg/m^2^)	−1.136	2.61	−.08	−6.44	4.16	.67		
eGFR	−1.01	0.59	−.29	−2.21	0.18	.10		
Matsuda index	0.88	2.32	.07	−3.82	5.59	.71	0.09	0.01
Step 4								
BMI (kg/m^2^)	−2.37	2.41	−0.16	−7.27	2.52	.33		
eGFR	−0.88	0.54	−.25	−1.97	0.211	.11		
Matsuda index	0.90	2.10	.07	−3.38	5.18	.67		
GIP/GLP-1 SR AUC	0.04	0.01	.44	0.10	0.06	.01*	0.27	0.18

This table presents unstandardized (B) and standardized (β) regression coefficients, SE, 95% CIs, and significance levels (*P*-values) for each predictor. The dependent variable was oral glucose tolerance test-derived standardized ISR1. GIP/GLP-1 AUC was the only significant predictor (*P* = .01), while BMI, eGFR, and Matsuda index were not significant. Bold values indicate statistically significant regression coefficients (*P < .05; **P < .01).

Abbreviations: AUC, area under the curve; BMI, body mass index; CI, confidence interval; eGFR, estimated glomerular filtration rate; GIP, glucose-dependent insulinotropic polypeptide; GLP-1, glucagon-like peptide-1; ISR1, insulin secretion at low glucose; LL, lower limit; SR, secretion ratio; UL, upper limit.

## Discussion

Our study demonstrates a dysfunctional incretin secretion pattern toward metabolic deterioration from normal glucose tolerance to overt diabetes. Specifically, we observed a reduced GIP/GLP-1 SR in diabetic individuals compared to prediabetic and normal glucose tolerant subjects, suggesting a disproportionality in the mutual dynamics of incretin secretion in T2D.

Regulation of the entero-insular axis and GIP and GLP-1 biological function is fundamental to maintaining appropriate glucose homeostasis in humans: this is particularly evident in T2D, where the loss of the incretin effect leads to ineffective coping to postprandial insulin demand (16). Many studies tried to investigate whether these defects could be the result of defective GIP and GLP-1 secretion, but evidence failed to identify a uniform GIP and GLP-1 secretion impairment in T2D (7, 8): this is in line with our findings, confirming similar absolute GIP and GLP-1 secretion in NGT and IGT subjects compared to newly diagnosed T2D patients without diabetes-related complications, thus excluding the possible impact of impaired gastric emptying rates as well as possible chronic kidney disease-related modifications of incretins clearance. By contrast, our investigation on the interrelationship between GIP and GLP-1 secretion—expressed in this study by the GIP/GLP-1 SR, assumed as an index of mutual incretins secretory dynamics in response to a mixed meal—revealed an imbalance in the overall incretin secretion, rather than in the single hormone per se, as confirmed by comparison of GIP/GLP-1 AUCs in the 3 groups and by linear mixed model analysis, contemplating the effects of glucose tolerance and time on hormones secretion.

Previous investigations on incretin pathophysiology have already established the impact of reduced islet sensitivity to incretins in determining the loss of their insulinotropic power in the natural history of T2D: we have demonstrated a reduced incretin effect in nondiabetic people, regardless of circulating GIP and GLP-1 levels, predicting diabetes onset after acute β cell mass reduction by pancreatoduodenectomy. This confirms the pivotal role of reduced incretin sensitivity of islets in detecting subjects more prone to develop hyperglycemia among nondiabetic subjects (9). In addition, administration of GLP-1 receptor antagonist (RAn) during OGTT in healthy subjects primarily affected the glucose excursions only in the early postload phase, while GIP RAn led to impaired normalization of glycemic excursion during the whole postload period; further, coinfusion of GIP and GLP-1 RAn together led to overall impaired glucose tolerance and reduced β cell function, confirming the importance of the synergy between GIP and GLP-1 action in determining appropriate insulin response, at least in a physiological setting. Importantly, incretins RAn resulted in increased levels of endogenous GIP and GLP-1, demonstrating an active incretin hypersecretory response, possibly compensating acute incretin resistance (17, 18). Moreover, we have recently demonstrated that, despite similar levels of circulating GLP-1, individuals with new-onset diabetes exhibited increased levels of intraislet intact GLP-1 compared to NGT and IGT subjects, possibly improving islet homeostasis in a paracrine manner during the deterioration of β cell function (19). Starting from these assumptions, we hypothesize that our findings could be the result of adaptive secretory incretin responses to reduced islet sensitivity to incretins, namely chronic incretin resistance. This imbalance may reflect an adaptive or pathological shift in enteroendocrine cell function as glucose tolerance deteriorates. In this scenario, the GIP/GLP-1 SR could represent an early marker of incretin dysfunction and glucose metabolism impairment regardless of insulin resistance levels, as demonstrated by noncorrelation with EHC-derived glucose uptake. This is even more consistent considering the robust association between the GIP/GLP-1 ratio and specific parameters of early β cell dysfunction. We have already demonstrated that reduced RS, reflecting the β cell ability to rapidly respond to changes in glucose levels during early insulin release, predicted diabetes appearance in a cohort of nondiabetic subjects undergoing acute β cell mass loss by pancreatoduodenectomy (15); in the present study, we observed a positive association between RS and GIP/GLP-1 SR, possibly expressing a predictive role of disproportionate incretin secretion on impaired first-phase insulin secretion. Conversely, we observed no association with βCGS and tISR, both expressing the effectiveness of insulin secretion during the early phase + late phase of MMT stimulation: this data together could indicate that unbalanced levels of incretins could correspond to a failure of their insulinotropic activity in sustaining early insulin release, while insulinotropic activity on late-phase insulin secretion is still preserved. This is in line with previous stimulation studies with concomitant GIP and GLP-1 infusion, where reduced GIP sensitivity was identified as the major factor responsible for ineffective late-phase insulin responses to glucose in T2D patients compared to healthy controls (despite a normalizing effect of GLP-1 infusion on late-phase insulin release), while both incretins were unable to restore early-phase insulin responses mostly in all forms of diabetes (5, 6).

In addition, we observed positive association with ISR1, an index of the effectiveness of insulin secretion at normoglycemic levels, expressing the possible impact of unbalanced incretin secretion on basal β cell functional reserve. This result should be regarded as an effect on the model-assessed relationship between insulin secretion and glucose concentration, where an increase in ISR1 at unchanged βCGS means an upward shift of the whole relationship, as already noted with GIP/GLP-1R activation, such as with dipeptidyl peptidase-4 inhibitors (20), exenatide (21), or liraglutide (22).

From a clinical perspective, this study confirms the unusualness of considering the endogenous levels of GIP and GLP1 secretion in the pathogenesis of T2D. Thus, only when the incretin hormones were relatively considered together, we were able to detect differences in β cell functional parameters, potentially identifying patients at risk of β cell dysfunction and subsequent progression to overt diabetes. Furthermore, it is important to clarify that incretin hormones share a complex regulatory mechanism with glucagon responses, with GLP-1 inhibiting and GIP stimulating glucagon responses in a glucose-dependent manner (1). Despite the possible bidirectional interaction between these hormonal pathways, we found comparable glucagon and insulin levels across our study groups: this may be attributed to multiple factors, including preserved β cell function of our participants and the use of a MMT containing amino acids and lipids—both known stimulators of glucagon secretion—compared to standard OGTT stimulation.

Current therapeutic strategies targeting incretin pathways have already shown efficacy in improving glycemic control. While the role of GLP-1 RAns appears well established, the role of GIP-based therapies remains less clear. Notably, dual GLP-1 agonist/GIP agonist and GLP-1 agonist/GIP antagonists have both been shown to enhance glucose tolerance and β cell function in individuals with T2D, highlighting the need for further investigation into their specific mechanisms of action. In this context, an effect of this molecule on the restoration of an appropriate endogenous GIP/GLP-1 interrelationship cannot be excluded and in addition may pose the background to design new interventions aimed at restoring balanced incretin responses in early T2D by improving incretin sensitivity rather than increasing circulating incretins levels (23-25). While this study provides novel insights on incretin pathophysiology by including the entire spectrum of the natural history of type to diabetes (from normal glucose tolerance to newly diagnosed T2D), undergoing a deep metabolic evaluation of incretin secretion patters and β cell function, some limitations must be considered. First, the study cohort comprised a relatively small sample size, which may limit the generalizability of our results; second, the cross-sectional design of our study precludes conclusions about causality between GIP/GLP-1 imbalance and β cell dysfunction. Longitudinal studies exploring the trajectory of GIP/GLP-1 SR during the progression from NGT to T2D could elucidate its predictive value as an early marker of β cell dysfunction as well as possible cause-effect mechanisms between incretin secretion and β cell impairment. Additionally, mechanistic studies focusing on the regulation of K- and L-cell function in T2D may uncover the underlying causes of the observed secretion imbalance.

In conclusion, our findings demonstrate that the GIP/GLP-1 SR declines as glucose tolerance deteriorates, reflecting an imbalance in mutual relative incretin dynamics rather than absolute hormone secretion. This imbalance may indicate early β cell dysfunction and chronic incretin resistance. The GIP/GLP-1 SR emerges as a potential marker for insulin secretion impairments, highlighting the need for therapies targeting incretin balance and sensitivity.

## Data Availability

Datasets generated and analyzed during the current study are available from the corresponding author upon reasonable request. Supplemental materials are available in Figshare at https://doi.org/10.6084/m9.figshare.28624877.v2.

## Study Approval

The study protocol (ClinicalTrials.gov NCT02175459) was approved by the Comitato Etico Fondazione Policlinico Universitario Agostino Gemelli IRCCS—Università Cattolica del Sacro Cuore (P/656/CE2010 and 22573/14), and all participants provided written informed consent, which was followed by a comprehensive medical evaluation.

Clinical Trial Registration Number: NCT02175459
